# Evaluation of anti-infective potencies of formulated aloin A ointment and aloin A isolated from *Aloe barbadensis* Miller

**DOI:** 10.1186/s13065-020-0659-7

**Published:** 2020-02-07

**Authors:** Addai-Mensah Donkor, Martin Ntiamoah Donkor, Ngmenpone Kuubabongnaa

**Affiliations:** Department of Applied Chemistry and Biochemistry, Faculty of Applied Sciences, University for Development Studies, Navrongo Campus, Navrongo, Ghana

**Keywords:** Antibacterial, Antifungal, *Aloe barbadensis* Miller, *Escherichia coli*, *Staphylococcus aureus*, *Pseudomonas aeruginosa*, *Klebsiella pneumoniae*, *Candida albicans*, *Talaromyces flavus*

## Abstract

**Introduction:**

Isolated bioactive components of plants or their raw extract are utilized as complementary or alternate remedy in copious illnesses. The current research was aimed at assessing the activity of aloin A isolated from *Aloe barbadensis* Miller and its formulated ointment against six (6) selected clinical isolates.

**Methods:**

The column chromatography was utilized in isolating aloin A from chloroform/methanol solvent polarity. The characterization of the isolated compound was performed by spectroscopy techniques corresponding to UV, IR, ^1^H- and ^13^C-NMR spectroscopy. It was formulated as ointment using polyethylene glycol (PEG) and both the ointment and the isolated compound were probed for in vitro antimicrobial activity.

**Results:**

Aloin A has been isolated from chloroform/methanol solvent mixture. The structure has been explicated as (10*S*)-10-β-d-glucopyranosyl-1,8-dihydroxy-3-(hydroxymethyl)-9(10*H*)-anthracenone(1*S*)-1,5-anhydro-1-[(9*S*)-4,5-dihydroxy-2-(hydroxymethyl)-10-oxo-9,10-dihydro-9-anthracenyl]-d-glucitol. The minimum inhibitory concentration (MIC) of the isolated aloin A on the pathogens ranged from 2.5 to 5.0 mg/ml and 0.32 to 5.0 mg/ml for both aloin A and the formulated ointment respectively. It was further revealed that the activity of aloin A showed dose dependence against all the test microorganisms. There was no significant difference in the activity of the drug against *K. pneumoniae*, *S. aureus*, *E. coli*, *C. albicans* and *T. flavus* (P > 0.05) when the concentration was raised from 2.5 to 5 mg/ml, however, there was significant difference (P ˂ 0.05) in activity against *P. aeruginosa*. The formulated ointment exhibited dose dependent activity against all test microorganisms. At low concentrations, the ointment showed no significant difference in diameter zone of inhibition against all test microorganisms (P > 0.05) except *P. aeruginosa* which exhibited a highly significant difference (P < 0.05).

**Conclusion:**

Both the isolated aloin A and its formulated ointment demonstrated substantial inhibition of growth of the pathogenic strains. These findings sturdily suggest that aloin A is a nascent drug that could be explored as skin and wound transmittable agent.

## Introduction

Currently, one significant problem in human health is the less efficiency of commercial antibiotics against several pathogenic bacterial and fungal isolates. Specifically emphasized in literature among few others is *Staphylococcus aureus*, a gram-positive bacterium from Staphylococcaceae family, and considered one of the world’s most important infectious agents triggering disease spates linked to food consumption, poorly treated wounds, and hospital-associated infections [1, 2]. *S. aureus* is frequently described as being responsible for a variety of human and animal diseases and its epidemiological significance is mainly due to their ability of becoming highly resistant to common antimicrobials [3–5] and to a lesser degree to some of the major types of antibiotics [6, 7]. In the last decades, evolution of resistance, for example, to methicillin, has become an enormous problem for treatment of *S. aureus* infections. *Candida albicans* is a member of the normal human microbiome. However, it is an opportunistic fungal pathogen associated with infections such as thrush, vaginal yeast infections and diaper rash [8]. One of the commonest causes of vaginitis among middle age women is attributed to *candida* infection. The predisposing factors include drug addiction, obesity, intake of birth control pills, pregnancy, antibiotic therapy, hormone therapy and diabetes mellitus. The mortality rate of *candida* infections is reported to be 40% [9, 10].

Thus, scientists have increased research programs to develop new and more effective antimicrobial molecules and several plants have been used in diverse ways to extract potential antimicrobial compounds. Different authors have shown that plants have naturally bioactive compounds that could act alone or in synergy with antibiotics against bacterial isolates [11, 12]. Throughout the world today, extensive research work are on-going regarding therapeutic applications of herbal plants which are of unlimited abundance and are believe to improve quality of life. One of the medicinal plants which is unique in terms of geographic distribution, species abundance and chemical composition is the aloe plant [13].

The aloe plant is known to have several species of which *Aloe barbadensis* Miller (*Aloe vera* L.) and *Aloe arborescens* are grown commercially [14]. Traditionally, *Aloe vera* gel is used for both external and internal applications. Topically, it has been used for treatment of wounds, minor burns and skin irritations and internally for constipation, coughs, ulcers, diabetes, headaches, arthritis and immune-system deficiencies [15]. Thus, we set this study in which we isolated and characterized aloin A and investigated its antimicrobial potential against different isolates of important pathogens highly associated with outbreak of diseases and antibiotic resistance phenomena.

## Methods

### Collection of plant material

Plant material, *Aloe barbadensis* Miller leaves, were purchased from Parks and Gardens in Kumasi in the Ashanti Region, Ghana. Officials followed the prescribed legislation and guidelines in collection and sale of the plant material. It was subsequently identified and authenticated by a plant taxonomist at the herbarium of Ghana Herbaria, Northern Savanna Biodiversity:Savanna Herbarium. The voucher specimen (number: SH 722) was deposited in the herbarium.

### Preparation of plant crude extracts

*Aloe barbadensis* Miller leaves were washed with distilled water and dried under sunshade for 4 weeks to ensure the leaves were devoid of moisture. The dried leaves were then pounded with the aid of mortar and pestle to obtain uniform powder, which was stored in an air-tight clean container. Powdered leaf sample (1.5 kg) was macerated in 5 l of absolute ethanol for 72 h at room temperature. The mixture was periodically shaken to enhance the extraction of the bioactive phytochemicals. The mixture was then filtered and the solvent was removed using rotary evaporator at temperature of 40 °C, to give a dark gummy residue. The yield was calculated to be 155.6 g (10.3% w/w).

### Isolation of aloin A by column chromatography

**Fig. 1:**
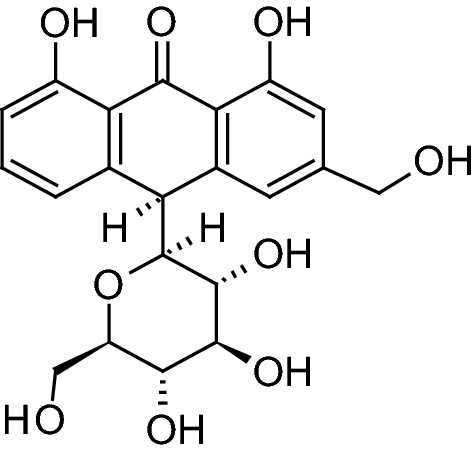
Structure of aloin A

Applying the method described by Coopoosamy and Magwa [16], extract (52 g) was partitioned between *n*-hexane and water. The *n*-hexane portion (5.5 g) was further fractionated by column chromatography over silica gel H (60–120 μm mesh size) using solvents of increasing polarity (0–100% EtOAc in *n*-hexane) and a total of 44 fractions were collected. The fraction obtained with 45–50% EtOAc in *n*-hexane was subsequently subjected to gel filtration (Sephadex LH-20), eluted with CHCl_3_ followed by MeOH:CHCl_3_ (5:95), producing a total of 30 fractions. Fractions 9–18, totaling 0.25 g, were combined and subjected to column chromatography over silica gel H, using solvent system CHCl_3_:MeOH (0–30%). A total of 22 fractions (20 ml each) were collected. Fractions 22–27 (pinkish color) were eluted with CHCl_3_:MeOH (90:10, v/v) to give a total of 30 fractions. Fractions 9–18 were combined (0.25 g) and further fractionated by column chromatography over silica gel H using CHCl_3_:MeOH (90:10 v/v). Major compound with minor impurity co-eluted at 90% chloroform as a yellow-powder and was separated using preparative TLC over silica gel F254 60 coated on glass plates 20 cm × 20 cm (solvent system: MeOH:CHCl_3_, 0.5:9.5) to afford the compound aloin A (Fig. 1), a total amount of 39.6 mg.

Aloin A was reconstituted in dimethylsulphoxide (DMSO) to obtain the desired concentrations of 20 mg/ml, 10 mg/ml, 2.5 mg/ml, 1.25 mg/ml, 0.63 mg/ml, 0.32 mg/ml, 0.16 mg/ml, 0.08 mg/ml and 0.04 mg/ml. Aliquots were stored at − 80 °C until needed.

The identity of the isolated aloin A was confirmed based on the following: The UV and IR spectra were recorded on Beckman DU-7400 and Perkin Elmer FT-IR spectrometers respectively. ^1^H and ^13^C (500 MHz) NMR spectra were recorded on a Bruker AMX 500 instrument with chemical shift data reported in parts per million (ppm) relative to the solvent used with field gradient BBI (inverse) probe.

### Formulation of aloin A-polyethylene glycol (PEG) ointment

Using a method described by Donkor et al. [17], polyethylene glycol 4000 (PEG 4000) and Polyethylene glycol 400 (PEG 400), 30 g each, was weighed into a beaker and melted on a thermostatic water bath at 45 °C until liquefied. It was then stirred with a glass rod under tap water at room temperature until congealed. Aloin A isolated from *Aloe barbadensis* Miller leaf extract was formulated with PEG ointment. The following quantities 20, 10, 5, 2.5, and 1.25 mg of aloin A were weighed into cleaned labeled separate beakers and 1.0 g of the PEG ointment was then added to each beaker and warmed at 70 °C while stirring continuously with a sterile glass rod for about 30 min. The mixtures were allowed to cool at room temperature to produce an aloin A‒PEG ointment at varying concentrations of 20 mg g^−1^, 10 mg g^−1^, 5 mg g^−1^, 2.5 mg g^−1^, 1.25 mg g^−1^.

### Test microorganisms

Clinical isolates of *P. aeruginosa*, *E. coli*, *K. pneumoniae*, *S. aureus*, *C. albicans* and *T. flavus* were used for the current study. They were obtained from the Microbiology Department of the Tamale Teaching Hospital, Northern Region, Ghana. Bacteria were maintained at temperatures between 2 and 8 °C on nutrient broth, and fungi at 4 °C on potato dextrose agar.

### Agar well diffusion assay

The method described by [18, 19] was used for the agar well diffusion assay.

### Test for antifungal activity

The method described by Suurbaar et al. [19] was applied to explore the antifungal activity of aloin A. The fungal spores were washed from the surface of agar plates with sterile 0.85% saline containing 0.1% Tween 80 (v/v). The spore suspension was adjusted with sterile saline to a concentration of approximately  1.0 × 10^7^ cfu/ml. The inocula were stored at 4 °C for further use. Dilutions of the inocula were cultured on solid potato dextrose agar to verify the absence of contamination and to check the validity of the inoculum.

### Inoculum preparation for minimum inhibitory concentration (MIC) and minimum bactericidal concentrations (MBC)

Using the method described by Suurbaar et al. [19], inoculum for the MIC and MBC test was prepared. At least three to five well isolated colonies of the same morphology from agar plate culture were taken. Using a sterile loop, the top of each colony was touched and the loop was transferred into a tube containing 5 ml of normal saline and then vortexed. The broth culture was incubated at 37 °C and monitored for approximately 4 h until it achieved the turbidity of 0.5 McFarland standard (1.5 × 10^8^ cfu).

### Determination of minimum bactericidal concentration (MBC) and minimum inhibitory concentration (MIC)

The method described by [18, 19] was employed for the determination of MBCs and MICs.

### Determination of minimum fungicidal concentration (MFC)

The method described by Donkor et al. [18] was used to determine the minimum fungicidal concentrations (MFCs). A commercial standard, Fluconazole (Sigma), was used as positive control (1–3000 μg/ml) and DMSO (99.9%) as negative control. All experiments were performed in triplicate and repeated two times for reproducibility.

### Statistical analysis

Means and standard deviation were calculated for the zones of inhibition measured for the two sets of experiments in each case. These means were statistically compared using One-way ANOVA followed by Tukey Multiple Comparison Test to determine if they were significantly different at P < 0.05.

## Results and discussion

### Isolation and identification of *Aloin A*

*Aloe barbadensis* Miller is native to Ghana and has been used by the indigenous people as a basis of therapy for several illnesses. There has been successful accomplishment of isolation and purification of many compounds from aloe species [20, 21]. The compound, aloin A (1,8-dihydroxy-3-hydroxymethyl-9-antracenone) (Fig. 1), was isolated from *n*-hexane/water branch extract of *Aloe barbadensis* leaf through chromatograph processes using different solvent systems with yield of 27%. Coopoosamy and Magwa [16] reported 30% yield of aloin A isolated from *Aloe excelsa*, using CHCl_3_:MeOH (90:10 v/v). Renuka et al. [22] used HPLC method and reported 37.2% yield of Aloin content with DimamonsilC-18 (250 mm × 4.6 mm 5 µl) column, methanol–water–acetic acid (42:58:0.5) mobile phase. Minale et al. [23] also reported 28.4% yield of Aloin A isolated from the leaf latex of *Aloe sinana* Reynolds using a mixture of chloroform and methanol (4:1). The varying yields could be attributed to the different species of the plant, the different portions of the leaf used, different solvent polarity and the method employed.

The spectral data (IR, ^1^H and ^13^C NMR chemical shift assignments) (Table 1) were consistent with the data reported by Coopoosamy and Magwa [16] who had previously isolated the compound from *Aloe excelsa*. Although it is not a novel compound, its formulation with polyethylene glycol (PEG) as an ointment against wound pathogenic microorganisms has been accomplished in this work for the first time.

**Table 1:** Spectral data of isolated compound, aloin A

Spectral technique	Data
IR (KBr, cm^−1^)	3436, 2923, 1684, 1384
^1^H NMR (CD_3_OD)	δ 7.47 (1H, t, J = 8.0 Hz, H-6), 7.03 (1H, s, H-4), 7.02 (1H, d, J = 8.8 Hz, H-5), 6.86 (1H, s, H-2), 6.83 (1H, t, J = 8.0, H-7), 4.64 (2H, d, J = 3.6 Hz, 3-CH_2_-OH), 4.56 (1H, s, H-10), 3.38 (1H, dd, J = 9.2, 2.0 Hz, H-1′), 3.01 (1H, t, J = 9.2, H- 2′), 3.23 (1H, t, J = 8.8 Hz, H-3′), 2.89 (1H, t, J = 8.8 Hz, H-4′), 2.91 (1H, m, H-5′), 3.54 (1H, dd, J = 1.6, 11.6 Hz, H-6′), 3.40 (1H, dd, J = 4.0, 9.6 Hz, H-6)
^13^C NMR (CD_3_OD)	δ 163.4 (C-1), 114.5 (C-2), 151.5 (C-3), 64.6 (3-CH_2_-OH), 119.2 (C-4), 119.9 (C-5), 137.0 (C-6), 116.8 (C-7), 162.9 (C-8), 195.6 (C-9), 45.9 (C-10), 117.7 (C-1a), 143.3 (C-4a), 146.6 (C-5a), 118.7 (C-8a), 86.7 (C-1′), 71.9 (C-2′), 80.0 (C-3′), 72.1 (C-4′), 81.7 (C-5′), 63.3 (C-6′)

### Antimicrobial studies of aloin A

**Fig. 2:**
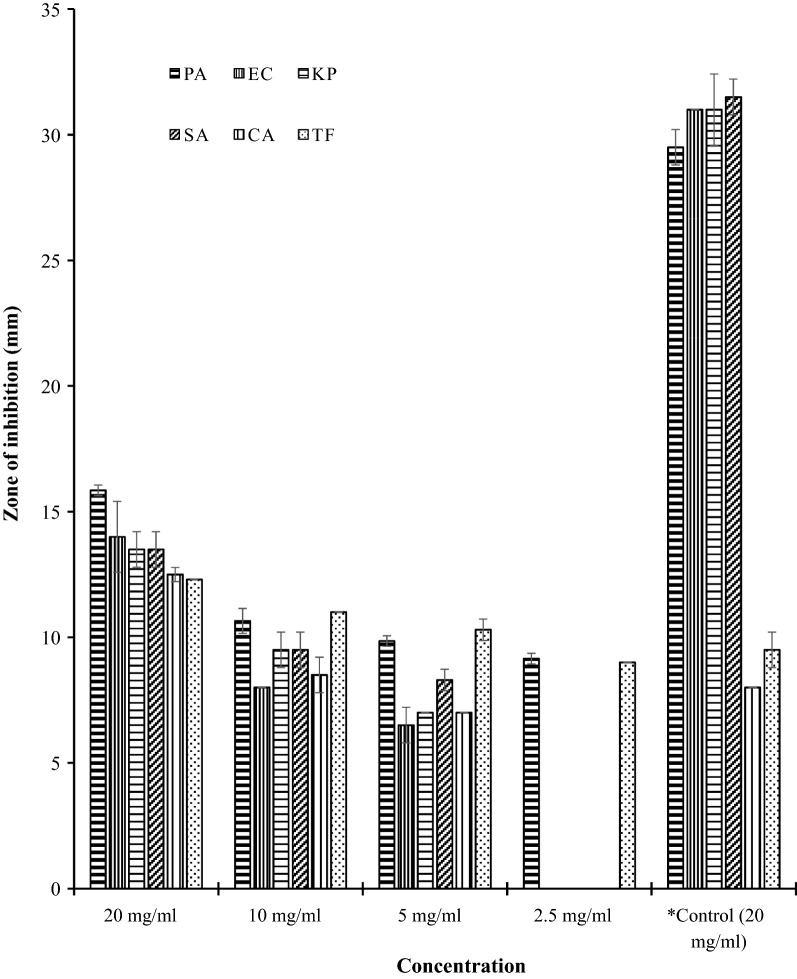
Antimicrobial activity of *aloin A*. *Control: chloramphenicol = +ve control for bacteria; fluconazole = +ve control for fungi. PA = *P. aeruginosa*; EC = *E. coli*; KP = *K. pneumoniae*; SA = *S. aureus*; CA = *C. albicans*; TF = *T. flavus*

In this research, the leaves were used because traditionally they have been used to treat wound and skin related diseases in Ghana. Antimicrobial activity of aloin A against the six (6) selected clinical isolates exhibited low MIC values (Table 2) revealing that the compound was effective against the entire microorganisms used in this research. All the test microorganisms showed high sensitivity towards the compound with maximum activity recorded against *P. aeruginosa* with zone of inhibition of 15.9 ± 0.2 mm at a concentration of 20 mg/ml, and low activity recorded for *E. coli*, *K. pneumoniae*, *S. aureus*, *C. albicans* and *T. flavus* with zones of inhibition 14.0 ± 1.4, 13.5 ± 0.7, 13.5 ± 0.7, 12.5 ± 0.3 mm and 12.3 ± 0.0 respectively (Fig. 2; Additional file 1). The observed results further revealed that the activity of aloin A showed dose dependence against all the test microorganisms. There was no significant difference in the activity of the drug against *K. pneumoniae*, *S. aureus*, *E. coli*, *C. albicans* and *T. flavus* (P > 0.05) when the concentration was raised from 2.5 to 5 mg/ml, however, there was significant difference (P ˂ 0.05) in activity against *P. aeruginosa*. Moreover, at 20 mg/ml, the drug showed significant higher activity against the two fungi compared with fluconazole, the positive control (Fig. 2; Additional file 1). However, the positive control (chloramphenicol) for the bacteria used in this research showed significant higher activity compared with aloin A but the negative control (DMSO—99.99%) showed no inhibitory activity. Minale et al. [23] recounted aloin A isolated from *A*. *sinana* exhibiting strong activity against the different strains of *E. coli*, *S. typhi Ty2*, *Shigella*, *S. aureus* and *V. cholera* and was found to be comparable to ciprofloxacin. They further stated the isolated compound showed broad spectrum antibacterial activity against both Gram-positive and Gram-negative bacteria. As stated by Asamenew et al. [24], aloin A which was isolated from *Aloe harlana* Reynolds also showed activity against *E. coli*, *S. typhi Ty2*, *Shigella* and *V. cholera* bacterial strains. *B. pumilus* and *B. subtilis* were found to be the most resistant bacterial strains to aloinoside—a precursor of aloin A—whereas aloin showed weak activity against these bacterial strains with MIC value of 200 µg/ml. Additionally, *E. coli* that causes a spectrum of disease ranging from diarrhea to the life threatening disease, hemolytic uremic syndrome, was also the most inhibited bacterial pathogen by aloinoside with MIC value of 10 µg/ml.

**Table 2:** MIC and MBC/MFC of aloin A and aloin A-PEG formulated ointment (mg/ml)

Test organism	Aloin A		Aloin A-PEG ointment	
	MIC	MBC	MIC	MBC
Bacteria				
*P. aeruginosa*	2.5	UD	2.5	UD
*E. coli*	5.0	UD	0.63	UD
*K. pneumoniae*	5.0	UD	0.63	UD
*S. aureus*	5.0	UD	0.32	UD

Test organism	Aloin A		Aloin A-PEG ointment	
	MIC	MFC	MIC	MFC
Fungus				
*C. albicans*	5.0	UD	0.32	UD
*T. flavus*	1.25	UD	1.25	UD

*PEG* polyethylene glycol, *MIC* minimum inhibitory concentration, *MBC* minimum bactericidal concentration, *MFC* minimum fungicidal concentration, *UD* undetected

There are contrasting reports in scientific literature concerning the toxicity of aloin. It is reported that aloin exhibited antitumor activity against experimental murine tumors with no harmful side effects on the host metabolism [25]. In another research, Celestino et al. [26] indicated that *A. ferox* resin, found to contain 33.5% anthranoid glycosides derivates expressed as aloin, was nontoxic when its laxative activity was investigated in vivo. On the contrary, animal studies have indicated aloin to have induced dose-related increased incidences and severities of mucosal and goblet cell hyperplasia in male rats [27]. Aloin is also suggested to exhibit antibacterial property for certain intestinal commensal bacteria and decreases butyrate-production in a dose-dependent manner [28]. These contradictory results could be attributed to the route of administration; the method of production which may result in the inclusion of other products, thus affecting the percentage purity of aloin; as well as in vitro or in vivo studies [29].

### Antimicrobial studies of formulated *Aloin A*-*PEG ointment*

We sought to test activity of aloin A–PEG ointment against the selected microorganisms using agar well diffusion method. Polyethylene glycol (PEG) is a biodegradable synthetic polymer of ethylene oxide units. PEG is reported to be highly biocompatible and non-immunogenic [30]. Its applications focus in majority on drug delivery and targeted diagnostics. PEG shows outstanding toxicological safety vis-à-vis acute and chronic oral toxicity, embryotoxicity or skin compatibility [31]. PEGs have been used for many years in cosmetics, foodstuffs [32] and the pharmaceutical industries. Extra significant assets of PEG is the solvent power for numerous substances that are sparingly soluble in water [33].

**Fig. 3:**
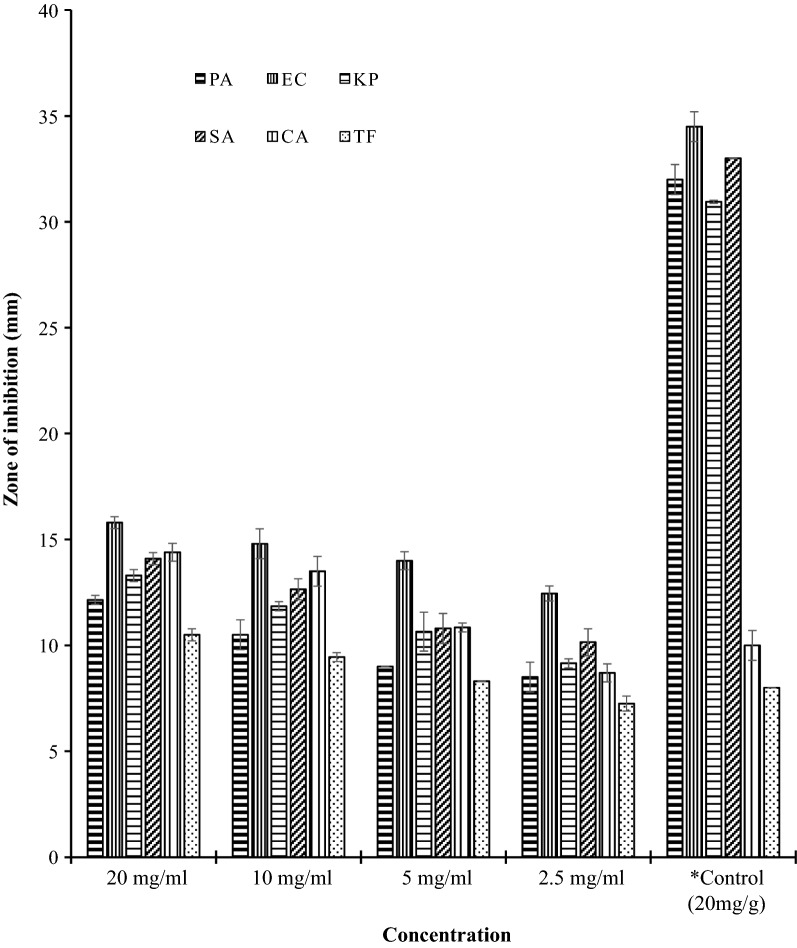
Antimicrobial activity of formulated aloin A ointment. *Control: chloramphenicol = +ve control for bacteria; Fluconazole = +ve control for fungi. PA = *P. aeruginosa*; EC = *E. coli*; KP = *K. pneumoniae*; SA = *S. aureus*; CA = *C. albicans*; TF = *T. flavus*

The antimicrobial effect of formulated aloin A-PEG ointment showed substantial activity against all the microorganisms with average zone of inhibition of 16 mm at 20 mg/g (Fig. 3; Additional file 2). The formulated ointment exhibited dose dependent activity against all test microorganisms. When the concentration of ointment was raised from 2.5 to 5 mg/g, there was no significant difference in diameter zone of inhibition against all test microorganisms (P > 0.05) except *P. aeruginosa* which exhibited significant difference (P < 0.05). However, at higher concentration there was highly significant difference (P < 0.05) in diameter zones of inhibition of all test microorganisms. Similar trends were recorded as the concentration of the ointment was increased up to 20 mg/g (P < 0.05). Similarly, the activity of aloin A alone, and its formulated ointment recorded significant activity against both fungi (*C. albicans and T. flavus*) than the positive control (fluconazole) used in this current research. However, chloramphenicol, positive control for the bacteria employed for this research expressed very high activity against *P. aeruginosa*, *E. coli*, *K. pneumoniae and S. aureus* than the formulated ointment (Fig. 3; Additional file 2).

The extensively spread of resistance to antimicrobial drugs is promoting revival in pursuit of new drug candidates for the treatment of diseases triggered by bacteria and fungi. Consequently, the in vitro activity of both aloin A and the aloin A–PEG ointment on the above disease causing bacterial and fungal isolates is highly substantial. Nonetheless, in vivo antimicrobial and toxicity studies need to be conducted before the products could be used in combatting bacterial and fungal infection.

## Conclusion

We have successfully isolated aloin A from the leaves of *Aloe barbadensis* Miller, characterized using ^1^H- and ^13^C-NMR as well as IR spectroscopy. We further tested the isolated compound alone and in combination with polyethylene glycol against some selected microorganisms such as *P. aeruginosa*, *E. coli*, *K. pneumoniae*, *S. aureus*, *C. albicans* and *T. flavus*. This research gives a scientific validation to the fact that aloin A, one of the bioactive components in *Aloe barbadensis* was extracted substantially in hexane/water/ethyl acetate mixture and exhibited highly promising bacterial and fungal inhibitory activity.

## Supplementary information

**Additional file 1.** Antimicrobial activity of aloin A (mm). The data represent diameter of inhibition of the compound against *P. aeruginosa*, *E. coli*, *K. pneumoniae*, *S. aureus*, *C. albicans* and *T. flavus*. The average of three wells treated on the same day was recorded. The experiment was repeated twice and day-to-day variation was found to be within one fold of the presented data.

**Additional file 2.** Antimicrobial activity of the formulated aloin A ointment (mm). The data represent diameter of inhibition of the formulated ointment against *P. aeruginosa*, *E. coli*, *K. pneumonia*, *S. aureus*, *C. albicans* and *T. flavus*. The average of three wells treated on the same day was recorded. The experiment was repeated twice and day-to-day variation was found to be within one fold of the presented data.

## Data Availability

All data generated or analysed during this study are included in this published article and its Additional files.

## Abbreviations

*A. vera*: *Aloe vera*
*K. pneumoniae*: *Klebsiella pneumoniae*
*E. coli*: *Escherichia coli*
*P. aeruginosa*: *Pseudomonas aeruginosa*
*S. aureus*: *Staphylococcus aureus*
*C. albicans*: *Candida albicans*
*T. flavus*: *Talaromyces flavus*
MIC: minimum inhibitory contration
MBC: minimum bactericidal concentration
MFC: minimum fungicidal concentration
SD: standard deviation
