# Supplementary material for: Evaluation of anti-infective potencies of formulated aloin A ointment and aloin A isolated from Aloe barbadensis Miller
Source: BMC Chem. 2020 Feb 7;14(1):8. doi: 10.1186/s13065-020-0659-7 (PMC7006150; doi:10.1186/s13065-020-0659-7)
Supplement: Supplementary file 2 — Additional file 2. Antimicrobial activity of the formulated aloin A ointment (mm). The data represent diameter of inhibition of the formulated ointment against P. aeruginosa, E. coli, K. pneumonia, S. aureus, C. albicans and T. flavus. The average of three wells treated on the same day was recorded. The experiment was repeated twice and day-to-day variation was found to be within one fold of the presented data. [file 13065_2020_659_MOESM2_ESM.docx]

**Additional file 2:** Antimicrobial activity of formulated aloin A ointment (mm)

| **Concentration** | ***P. aeruginosa*** | | | | ***E. coli*** | | | | ***K. pneumonia*** | | | | | ***S. aureus*** | | | | | ***C. albicans*** | | | | | ***T. flavus*** | | | | |
| --- | --- | --- | --- | --- | --- | --- | --- | --- | --- | --- | --- | --- | --- | --- | --- | --- | --- | --- | --- | --- | --- | --- | --- | --- | --- | --- | --- | --- |
|  | Test 1 | Test 2 | Mean | ± SD | Test 1 | Test 2 | Mean | ± SD | Test 1 | Test 2 | Mean | ± SD | Test 1 | | Test 2 | Mean | ± SD | Test 1 | | Test 2 | Mean | ± SD | Test 1 | | Test 2 | Mean | ± SD |  |
| 20 mg/ml | 12.0 | 12.3 | 12.2 | 0.2 | 16.0 | 15.6 | 15.8 | 0.3 | 13.5 | 13.1 | 13.3 | 0.3 | 14.3 | | 13.9 | 14.1 | 0.3 | 14.7 | | 14.1 | 14.4 | 0.4 | 10.3 | | 10.7 | 10.5 | 0.3 |  |
| 10 mg/ml | 10.0 | 11.0 | 10.5 | 0.7 | 15.3 | 14.3 | 14.8 | 0.7 | 11.7 | 12.0 | 11.9 | 0.2 | 13.0 | | 12.3 | 12.7 | 0.5 | 14.0 | | 13.0 | 13.5 | 0.7 | 9.6 | | 9.3 | 9.5 | 0.2 |  |
| 5 mg/ml | 9.0 | 9.0 | 9.0 | 0.0 | 14.3 | 13.7 | 14.0 | 0.4 | 10.0 | 11.3 | 10.7 | 0.9 | 11.3 | | 10.3 | 10.8 | 0.7 | 11.0 | | 10.7 | 10.9 | 0.2 | 8.3 | | 8.3 | 8.3 | 0.0 |  |
| 2.5 mg/ml | 8.0 | 9.0 | 8.5 | 0.7 | 12.7 | 12.2 | 12.5 | 0.4 | 9.0 | 9.3 | 9.2 | 0.2 | 10.6 | | 9.7 | 10.2 | 0.6 | 9.0 | | 8.4 | 8.7 | 0.4 | 7.0 | | 7.5 | 7.3 | 0.4 |  |
| *Control (20mg/g) | 32.5 | 31.5 | 32.0 | 0.7 | 34.0 | 35.0 | 34.5 | 0.7 | 30.9 | 31.0 | 31.0 | 0.1 | 33.0 | | 33.0 | 33.0 | 0.0 | 9.5 | | 10.5 | 10.0 | 0.7 | 8.0 | | 8.0 | 8.0 | 0.0 |  |

*Control: Chloramphenicol was used as positive control for bacteria; Fluconazole was used as positive control for fungi.

The data shown represent the average of three wells treated on the same day. The experiment was repeated twice and day-to-day variation was found to be within one fold of the presented data.
